# Joint metabolomic and transcriptomic analysis identify unique phenolic acid and flavonoid compounds associated with resistance to fusarium wilt in cucumber (*Cucumis sativus* L.)

**DOI:** 10.3389/fpls.2024.1447860

**Published:** 2024-08-07

**Authors:** Kankan Yang, Geng Zhou, Chen Chen, Xiaohong Liu, Lin Wei, Feiying Zhu, Zhihuai Liang, Huiming Chen

**Affiliations:** ^1^ Longping Branch, Graduated School of Hunan University, Changsha, China; ^2^ Hunan Academy of Agricultural Sciences, Changsha, China; ^3^ Hunan Vegetable Research Institute, Hunan Academy of Agricultural Sciences, Changsha, China

**Keywords:** cucumber, Fusarium wilt, metabolomic, RNA-seq, phenolic acid, flavonoids

## Abstract

**Introduction:**

Fusarium wilt (FW) caused by *Fusarium oxysporum* f. sp. *cucumerinum (Foc)* is a destructive soil-borne disease in cucumber (*Cucumis sativus*. L). However, there remains limited knowledge on the molecular mechanisms underlying FW resistance-mediated defense responses in cucumber.

**Methods:**

In this study, metabolome and transcriptome profiling were carried out for two FW resistant (NR) and susceptible (NS), near isogenic lines (NILs) before and after *Foc* inoculation. NILs have shown consistent and stable resistance in multiple resistance tests conducted in the greenhouse and in the laboratory. A widely targeted metabolomic analysis identified differentially accumulated metabolites (DAMs) with significantly greater NR accumulation in response to *Foc* infection, including many phenolic acid and flavonoid compounds from the flavonoid biosynthesis pathway.

**Results:**

Transcriptome analysis identified differentially expressed genes (DEGs) between the NILs upon *Foc* inoculation including genes for secondary metabolite biosynthesis and transcription factor genes regulating the flavonoid biosynthesis pathway. Joint analysis of the metabolomic and transcriptomic data identified DAMs and DEGs closely associated with the biosynthesis of phenolic acid and flavonoid DAMs. The association of these compounds with NR-conferred FW resistance was exemplified by *in vivo* assays. These assays found two phenolic acid compounds, bis (2-ethylhexyl) phthalate and diisooctyl phthalate, as well as the flavonoid compound gallocatechin 3-O-gallate to have significant inhibitory effects on *Foc* growth. The antifungal effects of these three compounds represent a novel finding.

**Discussion:**

Therefore, phenolic acids and flavonoids play important roles in NR mediated FW resistance breeding in cucumber.

## 1 Introduction

Cucumber, *Cucumis sativus* L., is an economically important vegetable crop. China is the dominant producer of cucumber, accounting for 70%-80% of total global production annually during the last decade (https://www.fao.org/faostat/en/#data/QCL). However, many diseases can impact the cucumber production, among which soil-borne fungal FW is the most difficult to control. This disease is particularly problematic in continuous cropping systems and protected environments. In China, under a continuous cropping system, the incidence of FW can range from 30% to 90% leading to significant yield loss (Zhou and Wu, 2012). The causal agent of this disease is *Fusarium oxysporum* f. sp. *cucumerinum* (*Foc*) (Owen, 1955). Typical symptoms of FW include yellowing, stunting, and the death of seedlings, as well as the yellowing and stunting of older plants. Infected plants wilt readily, lower leaves become yellow and dry, the xylem tissues turn brown, and the plant may die. These symptoms worsen when the plants experience stress or fruiting. This pathogen can survive in plant debris and soil for many years as chlamydospores (i.e., overwintering spores) and for shorter periods on greenhouse structures between crops as conidia. Large-scale, intensive cucumber production in protected environments such as greenhouses and high tunnels with infected soils increase the severity and frequency of FW, increasing difficulty in controlling this disease (Yu et al., 2000; Shen et al., 2008; Chen et al., 2012; Li et al., 2016).

Many integrated pest management (IPM) strategies have been proposed to control FW in cucumber, including deployment of resistant varieties, grafting, fumigation, crop rotation, and biological controls (Yu et al., 2000; Li et al., 2016; Tang et al., 2021; Nishioka et al., 2022). However, many of the proposed methods cannot be readily implemented in large-scale commercial production. The development of resistant varieties is likely the most economical and environmentally sound measure for the IPM of FW in cucumber production. In addition to reducing disease incidence and yield loss, resistant varieties can also improve the rhizosphere microbial community and soil quality (Yao and Wu, 2010). In China, several studies have evaluated FW resistance in cucumber collections (Mao et al., 2008b; Li et al., 2015, 2018). In a few cases, the FW resistance in different resistance sources was also characterized including the US cucumber inbred lines Wis248, WI2757, and WisSMR-18 (Netzer et al., 1976; Vakalounakis, 1993; Mao et al., 2008a, b; Vakalounakis and Lamprou, 2018), the cucumber germplasm line 9110Gt (Zhang et al., 2014), and the North China type cucumber lines Rijiecheng (Dong et al., 2019) and ‘3461’ (Bartholomew et al., 2022). The FW resistance in all these lines seems to be controlled by a single dominant gene. Moreover, the resistance gene *CsChi23* from ‘3461’ which encodes a cucumber class I chitinase with antifungal properties has been cloned (Bartholomew et al., 2022). Interestingly, molecular mapping studies suggest that the single domain resistance gene in all other cucumber lines is located in the same region on cucumber chromosome 2 that harbors a cluster of NB-LRR resistance gene homologs (Zhang et al., 2014). However, the identity and exact functions of this gene are unknown.

The molecular mechanisms of resistance gene mediated defense responses have been extensively studied and reviewed (Jones and Dangl, 2006; Spoel and Dong, 2012; Cui et al., 2015; Yuan et al., 2021; Aerts et al., 2022). Briefly, the establishment, penetration into the host cell wall and colonization of the host plant by the pathogens are facilitated by various enzymes such as pectinases, proteases, polygalacturonases and cellulases, which are secreted by the pathogens. Soilborne pathogens can stimulate plants to boost their defense mechanisms (Larkin and Fravel, 2002; Wen et al., 2023). To counteract pathogen attacks, upon infection, plant immune receptors recognize diverse pathogen molecules, leading to elicitor triggered immunity (ETI). ETI involves the activation of different biochemical pathways for the biosynthesis of pathogenesis-related (PR) proteins, callose formation, the accumulation of phytoalexins and cell wall modification that include lignification (Iqbal et al., 2021). The synthesis of various secondary metabolites such as flavonoids, catecholamines, phenolic acids, phenols, and lignins, plays an important role in disease defense responses (Dixon and Barros, 2019; Campos et al., 2021).

Nevertheless, how FW resistance genes regulate defense responses against *Foc* pathogen infection in cucumber remains largely unknown. Chitinases are pathogenesis-related (PR) proteins that were shown to play an important role in FW resistance (Bartholomew et al., 2019; Xu et al., 2021b; Bartholomew et al., 2022). Zhang et al. (2016) (Zhang et al., 2016) conducted comparative proteomic analysis of roots between two resistant lines and one susceptible cucumber line, and identified 15 over accumulated proteins that were involved in defense and stress responses, oxidation-reduction, metabolism, transport and other processes, as well as jasmonic acid and redox signaling components. Xu et al. (2021a) also compared the proteomes of the FW resistant Rijiecheng and FW susceptible Superina and identified 210 and 243 differentially regulated proteins in response to *Foc* infection with 32 predominantly expressed and significantly up-regulated in Superina after *Foc* inoculation. Dong et al. (2020) conducted transcriptome analysis in cucumber and suggested that ethylene-mediated defense responses play an important role in combatting *Foc* infection in cucumber.

Integrated omics (genomics, transcriptomics, metabolomics and proteomics) approaches have provided powerful tools for understanding the molecular mechanisms of resistance gene mediate defense responses in different crop plants (Kumar et al., 2016; Chen et al., 2019; Szymanski et al., 2020; Chai et al., 2021; Duan et al., 2022; Hussain et al., 2023). In this work, a highly inbred cucumber line (NR) with high resistance to FW was identified. Preliminary observations found that FW resistance in NR is controlled by a single domain gene. A spontaneous susceptible mutant plant (NS) was isolated from the resistant line (NR). In addition, RNA-Seq and widely targeted metabolomic analyses were conducted using two NILs. The objective of this study was to investigate the transcriptomes and metabolomes of the NILs to understand resistance gene mediated defense responses. The FW resistance of the NILs was evaluated, conducted of the transcriptomes and metabolomes were compared and integrated analyzed. Based on the results, the key genes associated with phenolic acids and flavonoid secondary metabolites that may play important roles in FW resistance in NR were identified.

## 2 Materials and methods

### 2.1 Plant materials and FW disease screening procedures

NILs of two south China ecotype cucumber, with contrasting FW inoculation, NR (high resistant) and NS (high susceptible), were used in the present study. These lines were provided by the cucumber research group of Hunan Vegetable Research Institute. A mutant NS high susceptible to FW was discovered in 2018 during the propagation of a high generation inbred line NR (resistance to FW) in a plastic greenhouse, which had continuously cultivated cucumber for over 23 years. After systematic breeding, 5 generations of self-homozygous mutant NS and 5 generations of NR were obtained.

The FW resistance of the NILs were evaluated in three growing seasons: autumn 2021, winter 2021, and spring 2022, using a randomized complete block design (RCBD) with 3 replicates and 15 plants per replicate. Inoculation of the FW pathogen (*Foc4.* There are 4 races of FW pathogen: *Foc1*: America, *Foc2*: Israel, *Foc3*: Japan, *Foc4*: China.) followed the national standard established by the department of Agriculture and Rural Affairs of China (NY/T 1857.3–2010; available at https://hbba.sacinfo.org.cn). Briefly, the seeds of the test cucumber lines were soaked in 5% sodium hypochlorite solution for 10 min, and washed with running water. The seeds were placed into a Petri dish with two layers of filter paper and kept in an incubator at 28°C for germination. Germinated seeds were planted in a sterilized seeding substrate with relative humidity of 75%, a temperature of 25°C/18°C, and a culturing cycle of 16 hour in day/8 hour at night. The *Foc* fungal strain was isolated from the cucumber roots exhibiting FW symptoms planted in the plastic greenhouse at Hunan Vegetable Institute, Hunan Province, China. For *Foc* inoculum preparation, the spores were propagated on potato dextrose agar (PDA) in plates at 28°C for 2 days, and then spores were harvested from culture in an incubator shaker at 180 rpm for 5 days at 28°C in potato dextrose broth (PDB), and the spore concentration was adjusted to 4×10^6^ spores mL^-1^. Seedlings in the first-true-leaf stage were inoculated via the irrigation method. It means the root length was measured at the 1st true leaf of the seedlings, and then they were planted in the plots containing equal amounts of sterilized soil and watered with equal amounts of water. Two days later, 5 mL of a 4×10^6^ concentration of spore solution was applied to each root. The root length of all seedlings was measured again at 4 dpi. FW symptom development was observed daily until 15 dpi, at which point the disease scores were used to calculate of the disease index (Zhang et al., 2014). All calculations of the significance of differences in our experiments were performed using two-way ANOVA (three data sets) in Graphpad Prism.

FW disease symptoms were rated with a 5-point scale (0–4) system: 0 = no symptom; 1 = cotyledon chlorosis but no wilting, 2 = cotyledon wilting, 3 = cotyledon and true leaf wilting or stunt seedling, and 4 = whole seedling withered (Lin et al., 2011). Disease index (DI) (Dong et al., 2019) was calculated using the follow equation:


$$\text{DI}=\frac{\sum \left(\text{Disease Grade x Corresponding Number of Pathogenic Seedlings}\right)}{\text{Highest Disease Grade x Total Number of Seedlings Investigated}}\times 100$$

The categorical assessment of FW resistance for each line was based on the DI, as follow: DI ≤ 10 = high resistance (HR), 10 ≤ DI ≤ 30 = resistant (R), 30 ≤ DI ≤ 50 = mediate resistance (MR), 50 ≤ DI ≤ 70 = susceptible (S), and DI > 70 = highly susceptible (HS).

Root and stem samples were taken from NS and NR (pooled from 15 plants) at 0 and 4 dpi, and labelled as NS0d, NS4d, NR0d, and NR4d, respectively. The samples were immediately flash frozen in liquid nitrogen and stored in a -80°C freezer for later use. The 12 samples were then divided into two parts for metabolomic and transcriptomic analyses.

### 2.2 Metabolomic analysis

Twelve samples (NS0d, NS4d, NR0d, and NR4d with three biological replicates each) were subjected to metabolomic analysis. The sample preparation, extract analysis, metabolite identification, and quantification were performed at Wuhan MetWare Biological Science and Technology Co. Ltd. (www.metware.cn) following standard protocol (Liu et al., 2020).

The pearson’s correlation coefficient r was used to assess the correlation between biological replicate. The pearson’s correlation coefficient was calculated using the built-in cor function in the R software (www.r-project.org/) (version V3.5.1): the more the |r| value toward 1, the better the correlation between replicate samples. PCA was performed using the built-in statistical prcomp function in the R software for PCA analysis, setting the prcomp function parameter scale as true to indicate that the data were subjected to UV (unit variance scaling) based on the calculated inter-sample euclidean distance. DAMs were identified based on the variable importance of the projection (VIP) ≥ 1 and | log_2_(fold change) | ≥ 1. Venn diagrams were used to illustrate the number of DAMs. The Kyoto Encyclopedia of Genes and Genomes (KEGG) database with a *p*-value of< 0.01 was used to study differentially metabolites in resistance to cucumber FW.

### 2.3 RNA-seq analysis

We conducted transcriptome profiling of the NILs with RNA-seq to explore the gene regulatory network associated with FW resistance. Total RNA was extracted from the 12 samples using an RNAprep Pure Plant Plus Kit (Tiangen, China). RNA quality was evaluated with a Nano Photometer spectrophotometer (IMPLEN, CA, USA) and an Agilent Bioanalyzer 2100 system (Agilent Technologies, CA, USA). Library preparation and Illumina sequencing were carried out with a device from Metware Biotechnology Co. Ltd. (Wuhan, China). High quality clean reads were mapped to the 9930 v2.0 reference genome (http://cucurbitgenomics.org/organism/2). Gene expression levels were determined using FPKM (Fragments per kilobase of transcript per million mapped reads). DEGs were identified through DESeq2 with a *p*-value of< 0.05 as the significance cutoff. GO enrichment and KEGG analyses of DEGs were performed with CuGenDBv2 (http://cucurbitgenomics.org/v2/).

### 2.4 Quantitation of total flavonoids and phenolic acids

Total flavonoid contents in NR and NS NILs were measured using a flavonoid content detection kit following the manufacturer’s protocols (Solarbio Biotechnology Co., LTD; https://www.solarbio.com/goods-6205.html). Flavonoids and aluminum ions formed a red complex in the alkaline nitrite solution with a characteristic absorption peak at 470nm. The flavonoid content was calculated by measuring the absorbance value of the sample extract at 470 nm. A standard curve was established by measuring the OD (optical density) from a dilution series of rutin trihydrate (CAS: 153–18-4) at 1.5, 1.25, 0.625, 0.3125, 0.15625, 0.078, 0.039, and 0.02 mg mL^-1^. The experiment was carried out using a standard tube and blank tube on the operation table. Based on the results, the OD value was measured and the standard flavonoid curve was drawn. The ΔA determination (y, ΔA determination) was entered the corresponding formula to calculate the sample concentration (x, mg mL^-1^).

The total phenolic acid contents in NR and NS were also measured using a total phenolic (TP) content detection kit from Solarbio. Under alkaline conditions, phenolic substances reduce tungstopolybdic acid and produce blue compounds with characteristic absorption peaks at 760 nm. The total phenolic content of the sample was obtained by measuring the absorbance value at 760 nm. The gallic acid (CAS: 149–91-7) standard solution was diluted to 0.625, 0.15625, 0.078125, 0.039, 0.02, 0.01, 0.005 and 0.0025 mg mL^-1^. The OD values for these solutions were draw the standard curve as a reference (Wang et al., 2020). (https://www.solarbio.com/goods-6204.html).

### 2.5 Quantitative real-time PCR

The RNA-Seq data were validated by using the qRT-PCR of eight selected DEGs. Gene specific primers were designed with Primer 5.0 (**Supplementary Table S9**). The CsActin gene was used as the internal reference. Total RNA was extracted using a TaKaRa MiniBEST Plant RNA Extraction Kit (TaKaRa Bio Inc., Kusatsu, Japan), and cDNA synthesis was performed using a TaKaRa PrimeScript™ RT reagent Kit with gDNA Eraser. PCR was carried out with TB Green Premix Ex Taq II (Tli RNaseH Plus) using TB Green Premix Ex Taq II (2×), 10.0 µL; forward primer (10 µM), 0.5 µL; reverse primer (10 µM), 0.5 µL; ROX reference dye (50×) 0.4 µL; DNA template, 1.0 µL; ddH_2_O 7.6 µL, per 20 µL reaction. The reaction was performed using a fluorescence quantitative PCR instrument (ABI 7300, Thermo Fisher Scientific, USA). The amplification and melting curves of the real-time PCR were confirmed after the reaction. The 2^-ΔΔCT^ method was used for calculations. Three biological replicates were used for each RNA sample.

### 2.6 Antifungal assays of selected DAMs

For requirements for a specific article type please refer to the Article Types on any Frontiers journal page. Lastly, two phenolic acids (bis (2-ethylhexyl) phthalate (117–81-7) and diisooctyl phthalate (27554–26-3)) and one flavone (gallocatechin 3-O-gallate (5127–64-0)) were used in an antifungal assay to study their effects on inhibiting the growth of *Foc* following the procedure of an early study (Ji et al., 2018) (Yuanye, Shanghai, China. https://www.shyuanye.com/index.html). Stock solutions of the three metabolites were dissolved in a *Foc* culture PDA medium with different concentrations: 0, 0.2, 0.4, 0.6, 0.8, and 1.0 g L^-1^. The concentration of the mixture included an equal volume of the three metabolites mixed at the same concentration. Next, 5 mm mycelium discs were taken from the colonies cultured for 7 days and placed in the center of a Petri dish (90 mm diameter) containing 15 mL PDA. The dish was incubated at 28°C and 90% relative humidity. The diameter of each colony was measured after 7 days, and the inhibition rates of the mycelial growth were calculated. Then, the linear model was applied to describe the relationship between the concentration and inhibition rate of three DAMs (Kong et al., 2023). The half-inhibition concentration (IC_50_) was calculated with the above models.

## 3 Results

### 3.1 *Foc* inoculation responses of near isogenic lines

**Figure 1:**
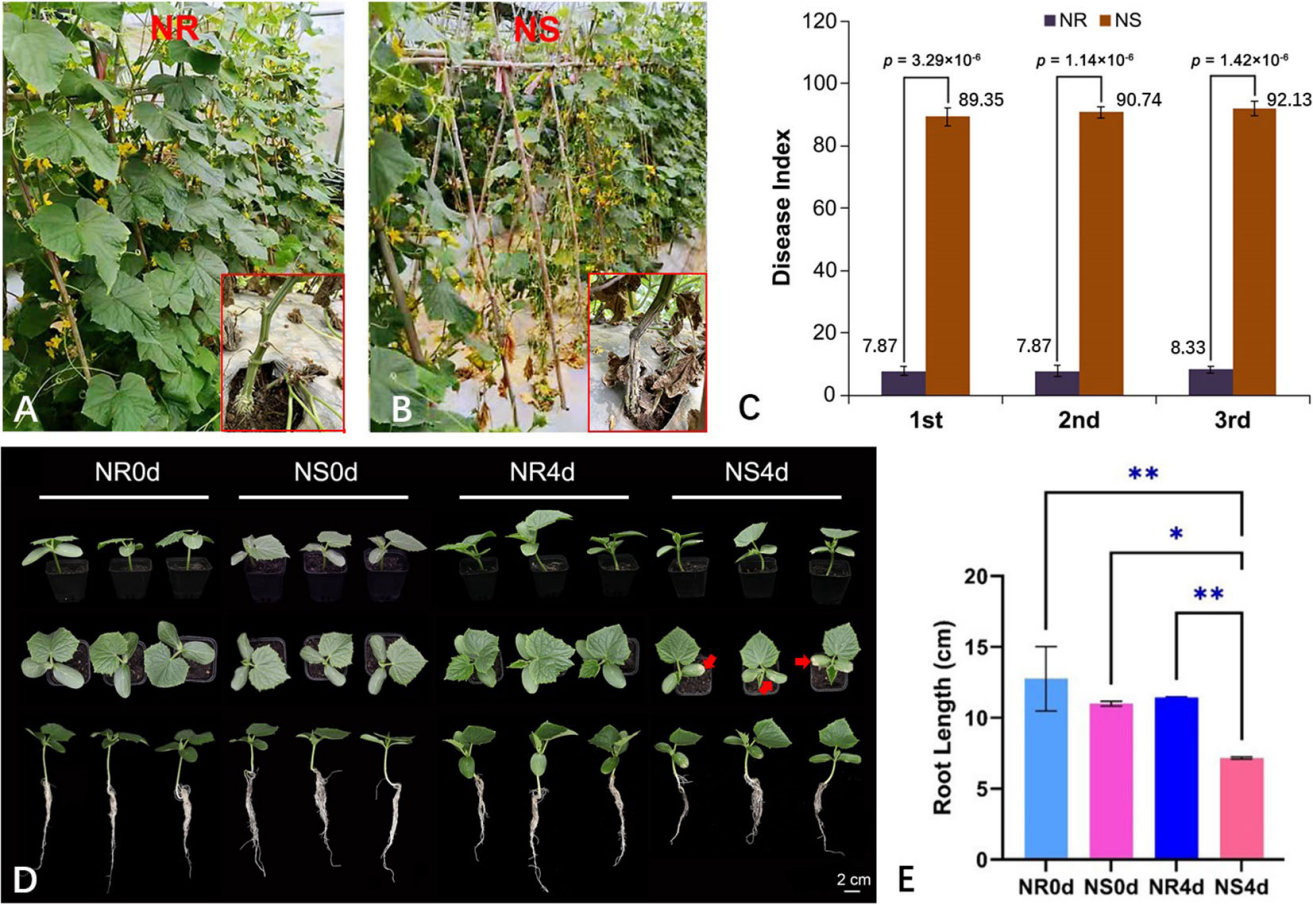
*Foc* inoculation responses in NR and NS NILs. NR **(A)** and NS **(B)** are highly resistant and susceptible to FW at adult plant stage in the greenhouse with *Foc* pathogen in the soil. In three experiments, disease index of NR is consistently lower than that of NS **(C)**. **(D)** At seedling stage, 4 days after *Foc* inoculation, chlorosis of the cotyledons and inhibition of root growth are clearly visible in NS but not in NR. The arrows are pointing to where the cotyledons are starting to turn yellow. **(E)** Root length of NR0d, NS0d, NR4d, NS4d. Data in **(C, E)** are based on three replications (n=15 in C and n=10 in E per rep), *p< 0.05, ** *p*< 0.01.

In this study, two highly inbred NILs, NR and NS, were developed to analyze FW resistance. To evaluate the FW resistance of the two NILs, we conducted replicated trials in a plastic greenhouse at the Hunan Vegetable Institute and in a plant greenhouse at the Hunan Institute of Plant Protection. An RCBD (randomized complete block design) experimental was designed (three replications, 15 plants per replicate) over three growing seasons: autumn 2021(2021A), winter 2021 (2021W) and spring 2022 (2022S). The plants were grown in the soil known to be infected with the *Foc* pathogen (**Supplementary Figure S1A**). Under natural infection, the NR plants consistently grew vigorously, while all NS plants exhibited typical stunted growth, wilting and eventually died of the *Foc* infection (**Figure 1A, B**). The stems of adult NR plants grew strongly and maintained a healthy greenish coloration while those from NS plants became dry and died (insets of **Figure 1A, B**). The disease index (DI) of NR and NS plants was calculated using the disease score at 15 days post infection (dpi) from all three experiments, as illustrated in **Figure 1C**. In all three seasons, the FW resistance of NR was highly stable with a mean DI of 8.02, while the NS was highly susceptible (mean DI = 90.73) (**Figure 1C**). Throughout all development stages, no morphological differences between the two NILs were visible, which was consistent with the near isogenic nature of the two lines. FW resistance in F1 plants from the cross between NR and NS was also observed. Resistance was high in all F1 plants (DI = 8.25) and in NR, suggesting that FW resistance in NR is the dominant factor in determining susceptibility.

The seedling-stage FW resistance of NR and NS with artificial inoculation was tested (3 replications, 10 seedlings per replicate). The chlorosis and root length of each seedling were recorded at 0 (before inoculation) (NR0d and NS0d) and 4 days post inoculation (dpi) (NR4d and NS4d). By 4 dpi, NS began to show stunted growth, and clear chlorosis on the cotyledons (**Figure 1D**, **Supplementary Figure S1B**). Root growth was also inhibited in NS seedlings (**Figure 1D**). The average root length of NR0d, NS0d, NR4d and NS4d was 12.76 cm, 11.00 cm, 11.44 cm and 7.17 cm, respectively (**Figure 1E**). The root length of NS4d was significantly shorter than that of NR4d, which was clearly caused by *Foc* infection (**Supplementary Figure S1A**). Therefore, the data indicated that the FW resistance conferred by NR was effective throughout all development stages.

### 3.2 Metabolomic analysis of NR and NS NILs in response to *Foc* inoculation

**Figure 2:**
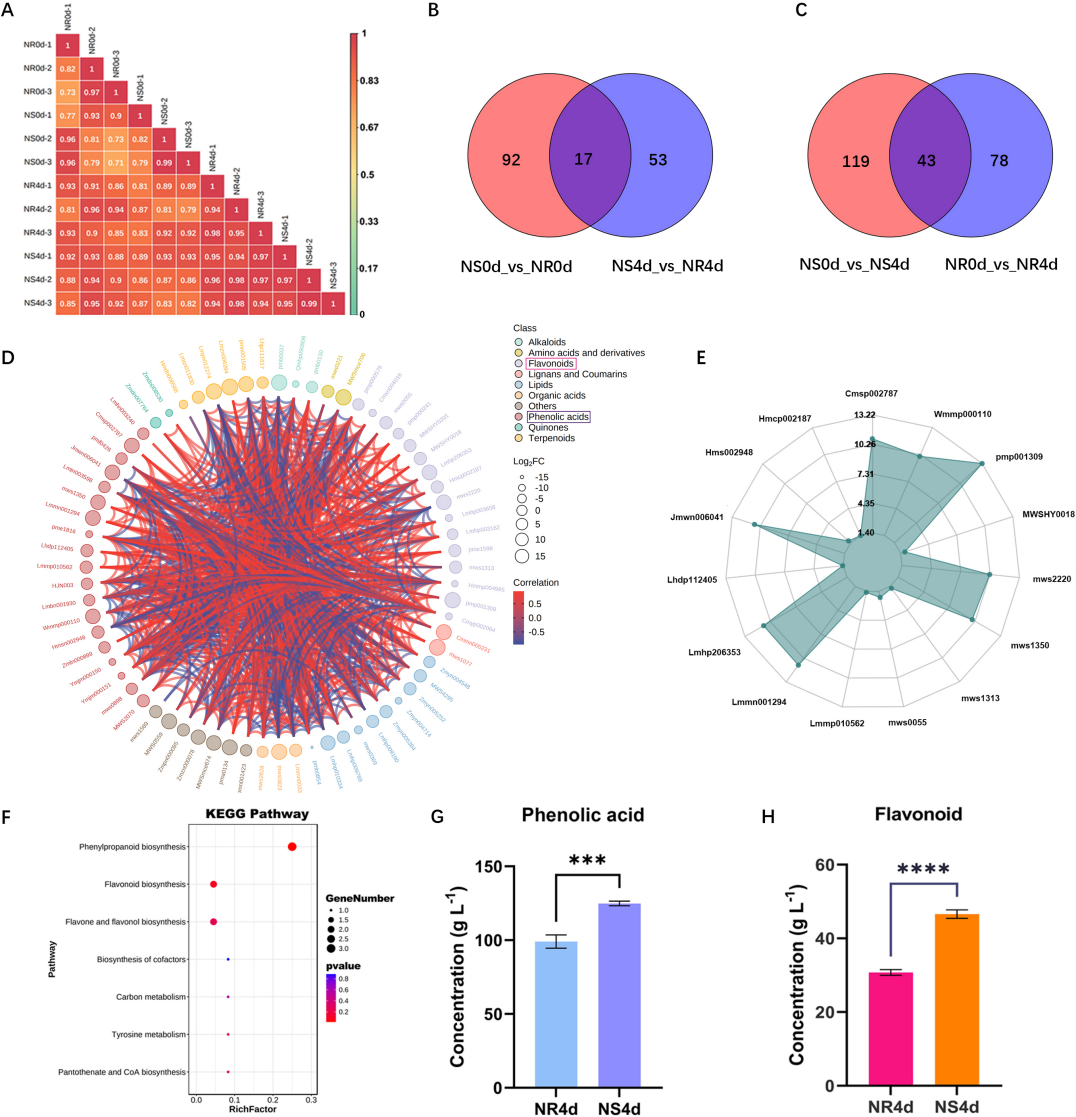
Targeted metabolomic analysis of NR and NS NILs identifies key metabolites in the flavonoid biosynthesis pathway associated with FW resistance. **(A)** Pearson’s correlation coefficients in pairwise metabolic data among biological repetitions and treatments indicating high reproducibility of data among replications of each sample. **(B, C)** Venn graph shows differential accumulated metabolite (DAMs) between NS0d_vs_NR0d and NS4d_vs_NR4d, NS0d_vs_NS4d and NSR0d_vs_NR4d comparison. **(D)** Chord diagram of DAMs in NS4d_vs_NR4d. **(E)** Radargram of phenolic acids and flavonoids in DAMs. **(F)** Enriched KEGG pathways of DAMs in NS4d_vs_NR4d comparison. **(G, H)** Total phenolic acids and flavonoids in NR4d and NS4d samples. Each data point is mean ± SD (n=3). *** and **** indicates *p<*0.01 and *p*<0.001 respectively from paired t-tests.

The seedling samples from the FW screening test described above were taken for targeted metabolome analysis which included two lines (NR and NS) and two time points (NR0d, NS0d, NR4d, and NS4d). Each sample included three biological replications (total 12 samples). Among the four samples, only NS4d showed significant symptom development, while the other three were considered symptom free (**Figures 1D**, **Supplementary Figure S1B**). Data output from the UPLC-MS/MS analysis were first subjected to data cleanup and conversion. The cleaned data were then further examined by performing correlation analysis. The pearson’s correlation coefficient plot for all 12 samples is shown in **Figure 2A**. This correlation coefficient was generally high with a mean of 0.90, suggesting low variation among different replications of the same sample and high reproducibility. As such, data from individual replications were pooled for each sample in all subsequent analysis.

Based on OPLS-DA (orthogonal partial least squares discriminant analysis), 925 metabolites were obtained in all samples. Using the criteria of VIP (Variable Importance in the Projection) ≥ 1, log_2_(fold change) ≥ 2 and *p*< 0.05, 283 differentially accumulated metabolites (DAMs) were identified base on the following four comparisons: NS0d_vs_NR0d, NR0d_vs_NR4d, NS0d_vs_NS4d and NS4d_vs_NR4d (NS0d as the control group, NR0d as the test group, the same for other comparisons). The last two comparisons were associated with different phenotypic responses upon *Foc* infection. Complete information on all 283 DAMs is presented in **Supplementary Table S1**. These metabolites could be classified into 11 categories: alkaloids, amino acids and derivatives, flavonoids, lignans and coumarins, lipids, nucleotides and derivatives, organic acids, phenolic acids, quinones, terpenoids, and others. Some categories could be further classified into subclasses (**Supplementary Table S1**). There were 121 total DAMs in NR0d_vs_NR4d, of which 87 and 34, respectively, showed a decrease and increase in NR4 (hereafter, we use the terms down-regulated and up-regulated for convenience). There were 162 DAMs in NS0d_vs_NS4d (78 down and 84 up-regulated), 109 in NS0d_vs_NR0d (26 down and 83 up-regulated), and 70 in NS4d_vs_NR4d (29 down and 41 up-regulated). There were 17 DAMs between NS0d_vs_NR0d and NS4d_vs_NR4d, and 43 DAMs between NS0d_vs_NS4d and NR0d_vs_NR4d (**Figure 2B, C**).

Since two comparisons NS0d_vs_NS4d and NS4d_vs_NR4d were associated with phenotypic FW symptoms in responses to *Foc* inoculation, subsequent analyses were focused on these two groups. The 70 DAMs between NS4d and NR4d included three alkaloids, two amino acids and derivatives, one Flavones, 16 flavonoids, two lignans and coumarins, two lipids, three organic acids, seven others, 19 phenolic acids, two quinones, 6 terpenoids, and seven unclassified (**Figure 2D**, **Supplementary Table S1**). The number of phenolic acids and flavonoids in this comparison accounted for almost 50% of total DAMs, suggesting their close association with FW resistance. Thirty DAMs in this category were identified, 50% of which were phenolic acids (8) and flavonoids (7) (**Figure 2E**, **Supplementary Table S2**). NR4d accumulated 2.82 times and 4.74 times more phenolic acids and flavonoids, respectively, than those in NS4d. Indeed, the KEGG pathway enrichment analysis indicated that these 30 DAMs featured an enriched synthesis of various secondary metabolites in the flavonoid biosynthesis pathway (**Figure 2F**, **Supplementary Table S3**). This result was validated through independent experiments. Total phenolic acids and flavonoids in NS4d and NR4d were measured, which revealed that the quantity of these two groups of compounds in NR4d was 1.27 times and 1.52 times higher than that in NS4d, respectively (**Figure 2G, H**). Therefore, the increased accumulation of phenolic acids and flavonoids may play a key role in conferring FW resistance in NR.

### 3.3 Transcriptome profiling of NR and NS NILs in response to *Foc* inoculation

**Figure 3:**
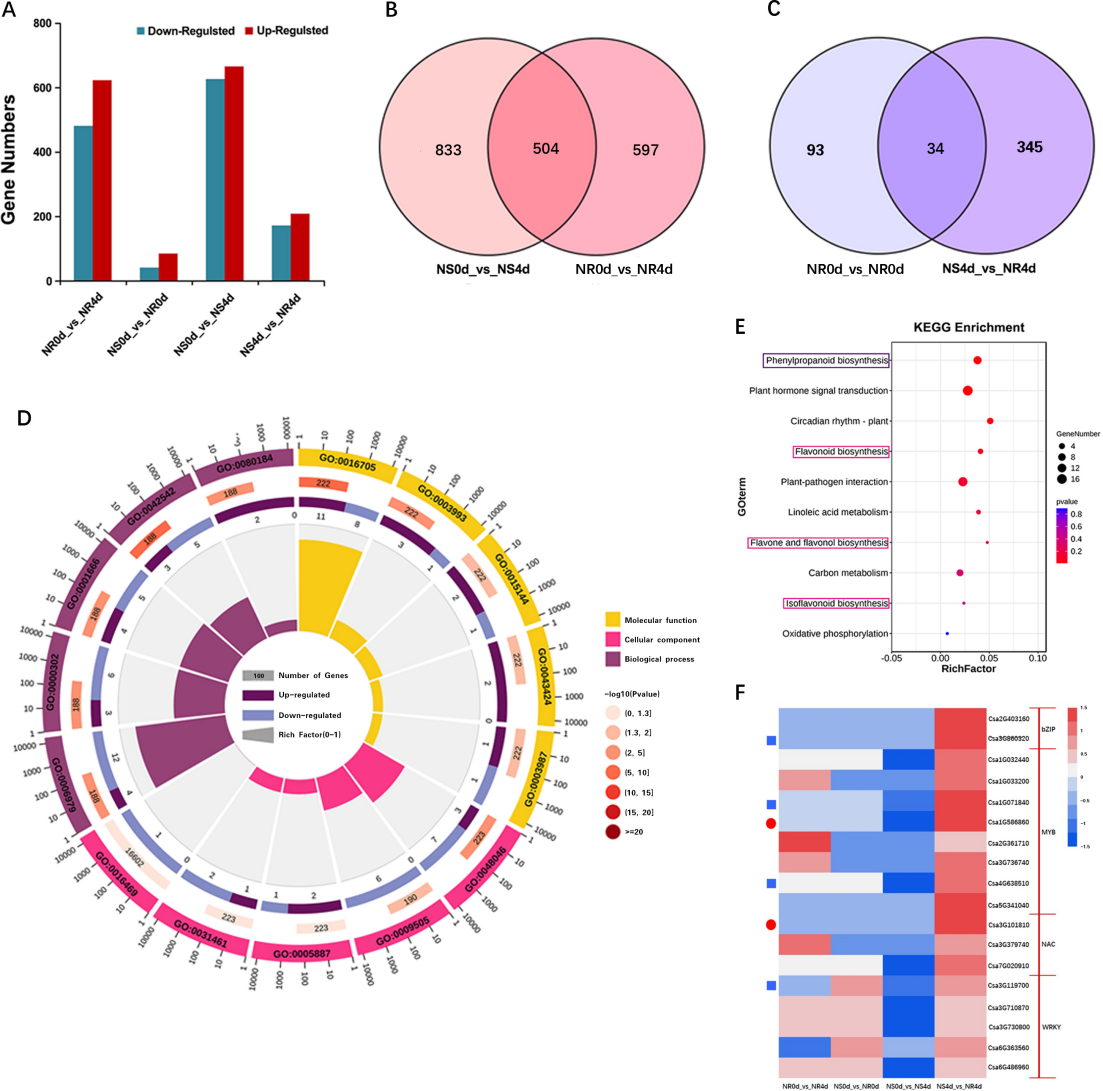
Transcriptome analysis of NR and NS NILs. **(A)** Bar graph of number of differentially expressed genes (DEGs) in different comparisons. **(B, C)** Venn diagrams show DEGs from com-parisons of different transcriptomes. **(D)** Enriched GO terms with DEGs in NS4d_vs_NR4d. **(E)** Enriched KEGG pathway terms from DEGS in NS4d_vs_NR4d. **(F)** Heat map of log_2_ (fold change) of selected DEGs. Blue rectangles and red circles indicate genes associated with phenolic acid and flavonoids biosynthesis, respectively.

It is well known that the accumulation of antimicrobe defense chemicals is due to resistance gene mediated transcription regulation of multiple biosynthetic genes. To understand - the regulatory network underlying the elevated accumulation of phenolic acids and flavonoids in NR, transcriptome profiling of the NILs was using the same set of 12 samples used for metabolomic analysis. High throughput Illumina sequencing generated approximately 81.6 Gb clean reads with Q20 and Q30 quality scores of 96.7% and 91.1%, respectively. Moreover > 92.74% of reads could be uniquely mapped to the cucumber 9930 v2.0 genome (**Supplementary Table S4**), indicating that the overall data quality was high. Using | log_2_ (fold change) | ≥ 1 and *p*-value< 0.05 as the threshold, differentially expressed genes (DEGs) were identified from four comparisons, which are presented in **Figure 3A**. There were 1101 (480 up-regulated and 621 down-regulated), 127 (42 up-regulated and 85 down-regulated), 1337 (624 up-regulated and 713 down-regulated), and 379 (172 up-regulated and 207 down-regulated) DEGs in the comparisons of NR0d_vs_NR4d, NS0d_vs_NR0d, NS0d_vs_NS4d, and NS4d_vs_NR4d, respectively. There were 504 DEGs between NS0d_vs_NS4d and NR0d_vs_NR4d, and 34 DEGs between NS0d_vs_NS4d and NS4d_vs_NR4d (**Figures 3B, C**). These data suggest the presence of very few constitutive differences in gene expression between NR0d and NS0d. However, significant changes occurred at 4dpi in both the NR and NS transcriptomes in response to *Foc* inoculation.

The expression patterns of selected DEGs were validated via qRT-PCR and primers for eight DEGs were designed (**Supplementary Table S5**), including *Csa1G043010, Csa4G620550, Csa6G084580*, and *Csa6G401340*, as well as four transcription factor (TF) genes that may be involved in regulating FW resistance: *Csa1G071840*, *Csa1G586860*, *Csa2G403160* and *Csa4G638510*. As shown in **Supplementary Figure S2**, the expression levels of *Csa1G043010*, *Csa4G620550*, *Csa6G084580* and *Csa6G401340* in NR4d were 2.47, 2.32, 2.14 and 2.89 times greater than those in NS4d, respectively. While the expression levels of the four TF genes were, respectively, 4.56, 2.47, 2.13 and 2.07 times greater. These data are consistent with those obtained via RNA-Seq, which further supports the reliability of the RNA-Seq data.

The DEGs from comparisons with samples featuring obvious wilt phenotype differences (NR0d_vs_NR4d, NS4d_vs_NR4d) were focused on. In total, 60 DEGs were selected out (**Supplementary Table S6**). The GO enrichment of DEGs in the combination with FW phenotype differences was compared (**Figure 3D**). Most DEGs in the Cellular component term belonged to apoplast (GO:0048046), plant-type cell wall (GO:0009505) and integral component of plasma membrane (GO:0005887). In the Biological process, DEGs were enriched in GO terms such as responses to phenylpropanoid (GO:0080184), oxidative stress (GO:0006979) and reactive oxygen species (GO:0000302). DEGs in the Molecular function term were enriched in oxidoreductase activity, acting on paired donors, with incorporation or reduction of molecular oxygen (GO:0016705), acid phosphatase activity (GO:0003993) and protein histidine kinase binding (GO:0043424) (**Supplementary Table S7**). KEGG pathway enrichment analysis showed that DEGs in the NS0d_vs_NS4d and NS4d_vs_NR4d comparison were concentrated in pathways for the biosynthesis of phenylpropanoids, flavonoids, flavone and flavonol, and isoflavonoids (**Figure 3E**, **Supplementary Table S8**). These data were highly incongruent with the results from the metabolic analysis.

In addition, 1770 transcription factors (TFs) were detected, 182 of which were identified to be significantly differentially expressed between NS and NR. The TFs acting against FW were mainly enriched in the MYB, WRKY, NAC and bZip transcription families. Further studies showed that one WRKY transcription factor (*Csa3G119700*), one bZip transcription factor (*Csa2G4031600*) and two MYB family transcription factors (*Csa1G071840, Csa4G638510*) may be related to phenolic acid metabolites. A NAC transcription factor (*Csa3G101810*) and MYB transcription factor (*Csa1G586860*) were associated with flavonoid metabolite synthesis (**Figure 3F**, **Supplementary Table S8**). It is speculated that these transcription factor coding genes may play an important role in the regulatory mechanism of resistance to FW.

### 3.4 Integrated analysis of metabolome and transcriptome data

**Figure 4:**
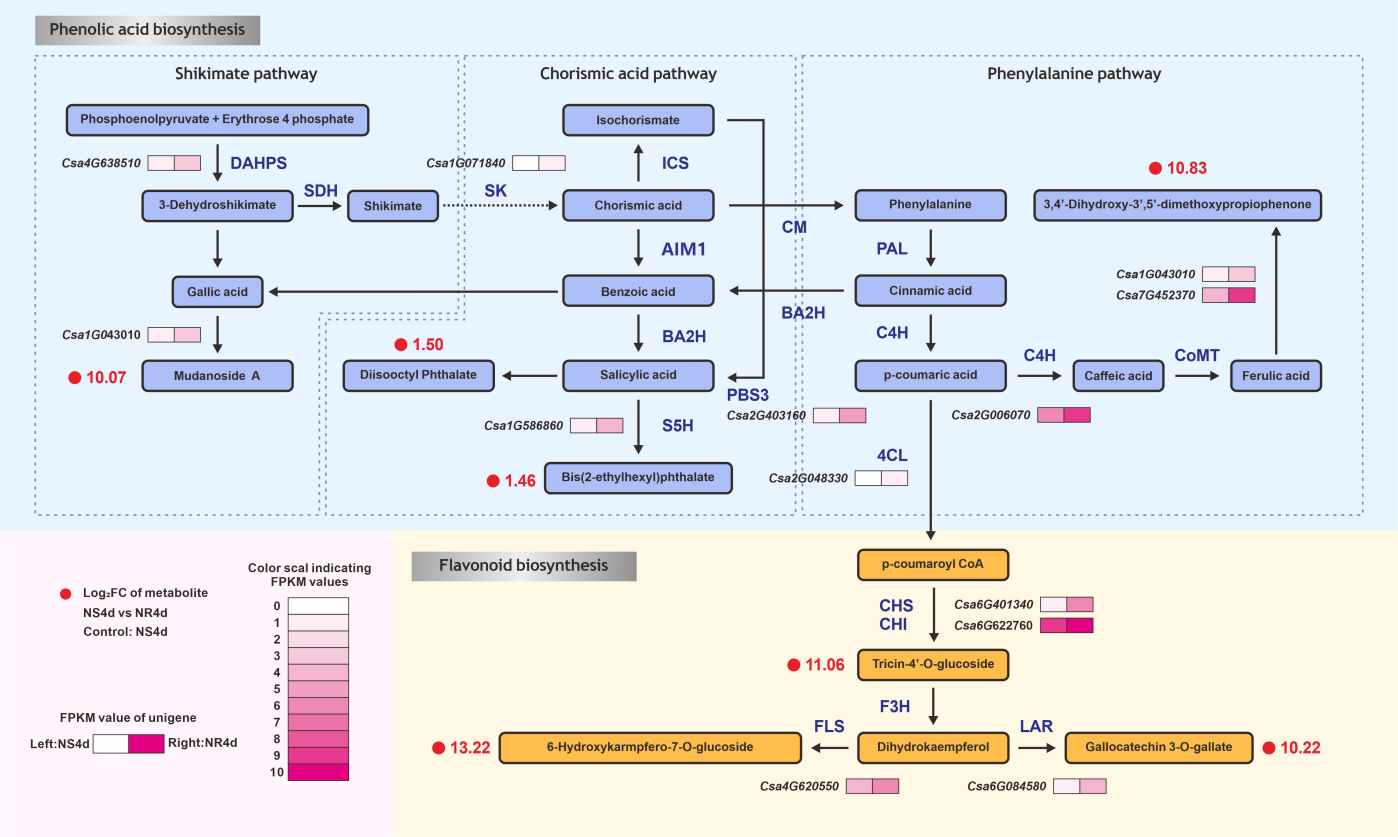
DEGs and DAMs in the comparison between NS4d vs NR4d that are mapped in the phenolic acid and flavonoid metabolic pathways. DAHPS: 3-deoxy-D-arabino-heptulosonate-7-phosphate hikimate dehydrogenase. SK, shikimate kinase; ICS, isochorismate synthase; AIM1, absent in melanoma 1 BA2H, benzoic acid 2-hydroxylase; PBS3, avrPphB susceptible; S5H, salicylic acid 5-hydroxylase; CM, chorismate mutase; PAL, phenylalanine ammonia-lyase; C4H, cinnamate-4-hydroxylase; C3H, coumarate-3-hydroxylase; CoMT, caffeic acid O-methyltransferase; 4CL, 4-coumarate-CoA ligase; HCT, hydroxycinnamoyl transferase; CHS, chalcone synthase; CHI, chalcone isomerase; F3H, flavanone-3-hydroxylase; FLS, flavonol synthase; LAR, leucoanthocyanidin reductase.

To further understand the relationships between DAMs and DEGs related to FW resistance in NR and NS NILs, the association of DAMs and DEGs was examined based on pearson’s correlation coefficients (r). Using the criteria of |r| > 0.8 and P< 0.05, 27 DAMs were found to be associated with 95 DEGs. These 27 DAMs included seven phenolic acids associated with 14 DEGs (**Supplementary Figure S3A**, **Supplementary Table S10**) and six flavonoids associated with 14 DEGs (**Supplementary Figure S3B**, **Supplementary Table S11**). These data suggested that metabolites were strongly correlated with the corresponding differentially expressed genes. Based on the correlation network analysis, the DAMs and DEGs from the NS4d_vs_NR4d were mapped to the flavonoid biosynthesis pathway (starting from shikimic acid), as shown in **Figure 4**. Through the metabolic analysis, seven phenolic acid metabolites and six flavonoids were identified (**Supplementary Tables S10, S11**). Several DEGs between NR4d and NS4d encode critical enzymes in this pathway. For example, chalcone synthase (CHS) is a rate-limiting enzyme in the biosynthesis of flavonoids. The CHS (*Csa6G401340*) presented higher expression in NR4d (2.13 times) than in NS4d. Similarly, the expression of FLS (flavonol synthase, *Csa4G620550*) and LAR (leucine thiocyanide reductase, *Csa6G084580*) was significantly upregulated in NR4d, which values 2.16 and 2.21 times those in NS4d.

Many TFs were shown to play important roles in resistance gene-regulated flavonoid biosynthesis in response to pathogen infection [39, 40]. In this pathway, expression of the MYB transcription factor encoding gene (*Csa1G071840*) in NR4d was 4.56 times higher than in NS4d. Expression of the carboxylesterase (*Csa6G401340*) gene in NR4d was up-regulated by 2.89 times compared with that in NS4d. The bZip TF gene *Csa2G403160* controlled the production of jasmonic acid from benzoic acid, with the expression of this gene found to be high in NR and NS. The MYB transcription factor coding gene (*Csa1G586860*, 1.06) can regulate the synthesis of jasmonic acid into genetic acid esters.

### 3.5 Antifungal effects of selected phenotypic acid and flavonoid compounds on *Foc* growth

**Figure 5:**
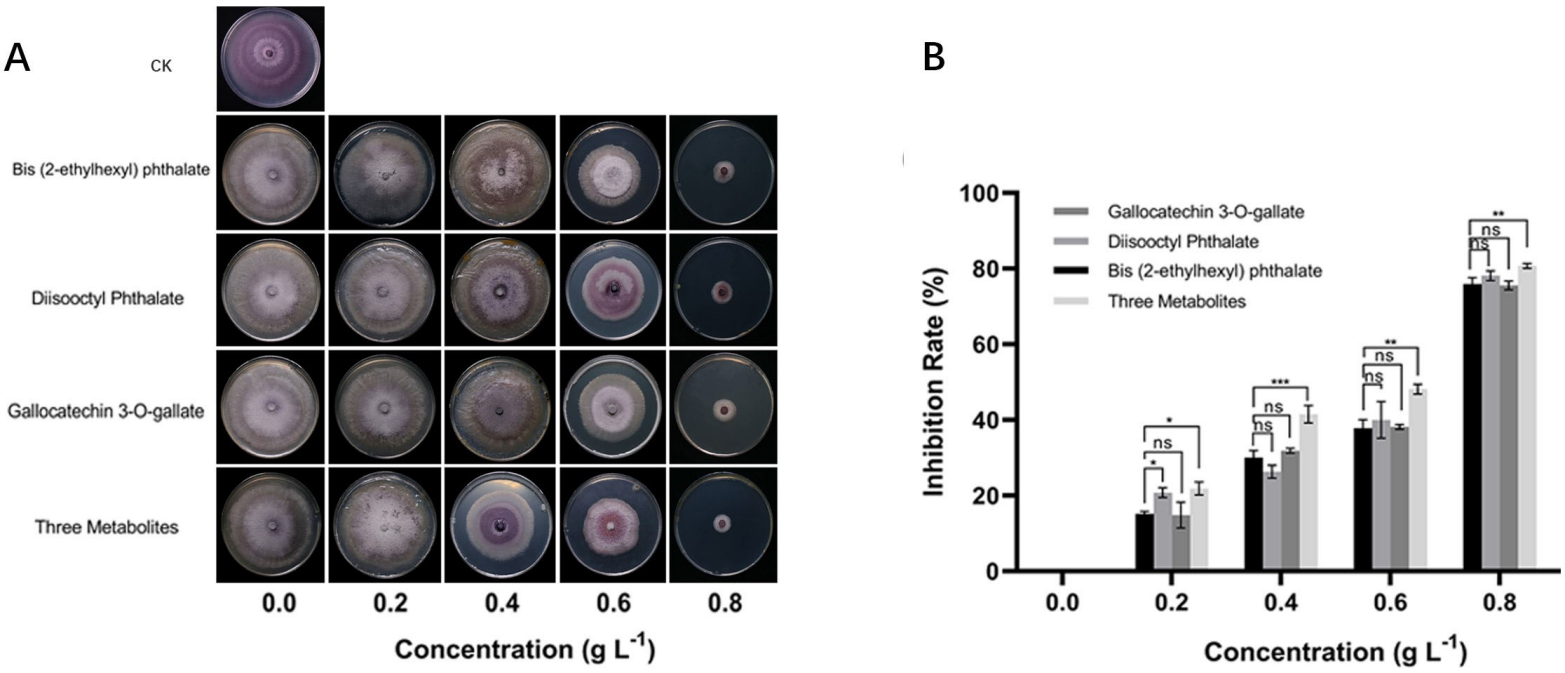
Bioassay of inhibitory effects of selected phenolic acid and flavonoid compounds on growth of *Foc* pathogen. **(A)** *In vitro* growth of *Foc* treated with different concentrations of bis(2-ethylhexyl) phthalate, diisooctyl phthalate, gallocatechin3-O-gallate and only PDA (CK) after incubation at 28°C for 7 days on PDA plate. **(B)** Inhibition rate of three chemicals and their combination on *Foc* growth. In B, each datapoint is mean ± SD (n=3). Asterisks (*) indicate the statistical significance of the difference between other compounds and the control group is bis (2-ethylhexyl) phthalate. * *p*< 0.05, ** *p*< 0.01, *** p< 0.001, and **** *p*< 0.0001, ns, not significant.

Compared to NS4d, NR4d accumulated more secondary metabolites in the flavonoid biosynthesis pathway suggesting that these compounds may contribute to the observed antimicrobial effects and thus resistance to FW. To confirm this phenomenon, we conducted an *in vivo* bioassay to evaluate the effects of selected DAMs on the growth of the *Foc* pathogen. Two phenolic acids, bis(2-ethylhexyl) phthalate and diisooctyl phthalate, and a flavonoid, gallocatechin 3-O-gallate, along with a mixture of all three were tested (**Figure 5**). For each chemical/mixture, there were six concentrations in the PDA growth media (0, 0.2, 0.4, 0.6, 0.8 and 1.0 g L^-1^) (**Figure 5A**). The *Foc* growth inhibition rate (**Figure 5B**) was measured at 7 days after culturing. All three compounds clearly inhibited growth of the pathogen. In each case, the inhibition rate and reduction of mycelial diameter were positively correlated with the concentration of the metabolites. The inhibitory effects of the three chemicals appeared to be additive because the inhibition of pathogen growth was most effective when using the mixture of the three compounds. For *Foc*, the IC_50_ (half maximal inhibitory concentration) of bis(2-ethylhexyl) phthalate, diisooctyl phthalate, gallocatechin 3-O-gallate and their mixture was 0.6628, 0.6636, 0.6786, and 0.5262 g L^-1^, respectively.

## 4 Discussion

### 4.1 The accumulation of phenolic compounds is positively correlated with FW resistance in NR

FW is a serious soilborne disease and is very difficult to control especially under continuous cropping in protected environments, which is popular in Chinese cucumber production (He et al., 2022). The development of host resistance is a critical component in the IPM of diseases in crop production. Although several resistance sources to FW have been identified (see introduction), most cucumber varieties in commercial production in China are FW susceptible. The only cloned FW resistance gene is the single dominant gene *Foc* from the north China inbred line ‘3461’, which encodes a class I chitinase (Bartholomew et al., 2019). Overall, the understanding of the molecular mechanisms underlying FW resistance in cucumber remains very limited. To test FW resistance in this study, NILs, NR and NS, were developed which belong to the South China cucumber type ecotype. In multiple screening tests in both plastic greenhouses (natural infection) and the laboratory (artificial infection), the NR sample exhibited consistent and stable FW resistance (**Figure 1**, **Supplementary Figure S1B**). Based on the results from the present study, this resistance gene is probably different from the *Foc* gene reported in line 3461 (Bartholomew et al., 2022).

To understand the gene regulatory network in resistance gene mediated defense responses to *Foc* infection in NR NIL, both metabolome and transcriptome profiling of the two NILs before (0 dpi) and after (4 dpi) *Foc* inoculation were using the same set of four samples (NR0d, NS0d, NR4d and NS4d) with three biological replicates. Many differentially accumulated metabolites (DMAs) and differentially expressed genes (DEGs) were identified between the resistant and susceptible NILs (**Figures 2**, **3**; **Supplementary Tables S1-S8**). Further analysis of the metabolomic data found a significantly greater accumulation of phenolic compounds (phenolic acids and flavonoids) in NR than in NS in response to *Foc* infection (**Figure 2**). Consistent with this result, DEGs response to the biosynthesis or regulation of secondary metabolite biosynthesis in the flavonoid biosynthesis pathway were highly enriched (**Figure 3**; **Supplementary Tables S6, S7**). These data strongly suggest that accumulation of phenolic compounds from the phenylpropane and flavonoid biosynthesis pathways contribute to FW resistance in NR.

Phenolic compounds are secondary metabolites in plants and may include phenolic acids (e.g., chlorogenic acid, caffeic acid, phydroxybenzoic acid, ferulic acid, 4-coumaric acid and gallic acid), flavonoids (e.g., flavanones, flavonols and proanthocyanidins), tannins, stilbenes, and lignans which are mainly synthesized by the shikimic acid, chorismate and phenylalanine metabolic pathways (Palacio et al., 2012; Dixon and Barros, 2019). Phenolic compounds have diverse functions in plant growth and development, reproduction, and defense. Such compounds may act as antioxidants, structural polymers (lignin), attractants (flavonoids and carotenoids), UV screens (flavonoids), signal compounds (salicylic acid and flavonoids) and defense response chemicals (tannins and phytoalexins) (Weisshaar and Jenkins, 1998; Dicko et al., 2005; Visioli et al., 2011; Dixon and Barros, 2019; Lima et al., 2019). The roles of phenolic compounds in plant disease resistance have been extensively documented (Kneusel et al., 1989; Tsuji et al., 1992; Dixon and Barros, 2019). For example, phenolic acids are key components of plant resistance to different pathogens (bacteria, fungi and viruses) (Mandal et al., 2010; Boiteux et al., 2014; Zhang et al., 2022). Upon pathogen infection, the plant may accumulate large amounts of phenolic acids (Mikulic Petkovsek et al., 2013). Phenolic acids are key signaling molecules that can be released rapidly from new roots during seed germination and seedling growth and may contribute to soilborne pathogens (Ndakidemi and Dakora, 2003; Mandal et al., 2010). Microbial changes influenced by signals from phenolic acids may also have ecological effects on plant-microbe interactions (Shaw et al., 2006). The accumulation of flavonoids is one of the general defense responses in plants (Ndakidemi and Dakora, 2003; Theunis et al., 2004; Li et al., 2011; Roy et al., 2018). The antifungal effects of flavonoids mainly included inhibiting the growth of fungal colonies, spore germination, and bud tube length (Xu et al., 2022). Therefore, data from the present study were consistent with previous findings and support the critical role of phenolic acids in defenses against *Foc* infection.

### 4.2 Key phenolic acids. flavonoids, and biosynthetic and regulatory genes associated with FW resistance

Joint analysis of the metabolomic and transcriptomic data using pearson’s correlation coefficients identified important associations between 13 DAMs (phenolic acids/flavonoids) and 20 DEGs (**Figure 4**; **Supplementary Figure S3**; **Supplementary Tables S10, S11**). Mudanoside A is one of the end products of phenolic acid compounds with gallic acid as the precursor of its synthesis. Mudanoside A catalyzes a hydrolase that appears to be associated with the function of *Csa1G043010* (**Figure 4**), which is involved in the biosynthesis of tulitin, a defensive chemical with antibacterial activities against a variety of bacterial and fungal strains (Lima et al., 2019; Milinčić et al., 2021; Zhu et al., 2022). Gallic acid concentrations were increased in infected chickpea leaves and stems compared to healthy plants (Sarma and Singh, 2003). Hydrolase coding gene *Csa1G043010* hydrolyzes ferulic acid to produce the phenolic compounds 3,4’-Dihydroxy-3’,5’-dimethoxypropiophenone, which can hydrolyze caffeic shikimic acid into caffeic acid and shikimic acid. Ferulic acid not only inhibits the growth of anthrax, but also prevents infections by *S. rolfsii* in the stems and leaves of chickpea plants, and it can also regulate plant root growth (Mandal et al., 2010) (Sarma and Singh, 2003; Zhang et al., 2022). It is speculated that the high expression of these genes in the regulation pathway of phenolic acid synthesis may lead to the accumulation of these phenolic acid compounds, thereby improving the resistance of plants to FW.

The synthesis of flavonoids in plants is a complex metabolic process controlled by a series of enzymes that varies according to species and tissues (Mikulic Petkovsek et al., 2013; Sade et al., 2015). In the present study, four flavonoid DAMs that accumulated in significantly higher quantities in NR were identified, including one intermediate product, tricin-4’-O-glucoside, and two end-products, 6-hydroxykaempfero-7-O-glucoside and gallocatechin 3-O-gallate (**Supplementary Table S11**). Three differentially expressed genes were strongly correlated with these products, namely, the carboxylesterase gene (*Csa6G401340*), UDP glycosyltransferase gene (*Csa4G620550*), and plant disease resistance protein gene (*Csa6G084580*). The chalcone synthase gene (CHS) (*Csa6G401340*) was up-regulated 2.13 times more strongly in NR4d than in NS4d, and is the first key gene in the phenylpropane biosynthesis to flavonoid biosynthesis. CHS regulates the formation of Tricin-4’-O-glucoside, which is a plant born antioxidant flavonoid that enhances the disease resistance of plants by improving their antioxidant activities (Zhong et al., 2022). Flavanols, flavonols and flavones are important subclasses of flavonoid compounds (Chen et al., 2021). The FLS (*Csa4G620550*) flavonol synthase coding gene was significantly upregulated in NR4d by 2.16 times compared with NS4d. This gene encodes UDP glycosyltransferase and catalyzes the formation of flavonol glucosides (Li et al., 2021). LAR (*Csa6G084580*) was 2.12 times more prevalent in NR4d than in NS4d, and associated with the synthesis of the plant disease resistance response protein. In flax, three key genes of flavonoids, CHS, CHI and DFR, were synthesized via the transgenic method, resulting in a significant increase in the contents of flavanones, flavonoids and flavanols, as well as increased flax resistance to Fusarium acarium (Lorenc Kukuła et al., 2007).

In addition to biosynthetic genes, many DEGs for transcription factors (TFs) were also identified via RNA-Seq (**Supplementary Table S9**). TFs, especially MYB and bZip TFs were found to play important roles in regulating phenolic compound accumulation to support disease resistance (Nuruzzaman et al., 2013; Shim et al., 2013; Liu et al., 2016; Jin et al., 2017; Bartholomew et al., 2022; Tang et al., 2022). For example, MYB TFs plays an important role in plant defense response to biological stress, which triggers a wide range of plant defenses. SpMYB (Solanum pimpinelifolium L3708) was significantly expressed in tobacco after infection with *fusarium oxysporum*. The overexpression of SpMYB in tobacco increased resistance to *Fusarium oxysporum*, and *peroxidase*, *superoxide dismutase* and *phenylalanine* increased the activity of ammonia lyase in transgenic plants (Wang et al., 2015; Xie et al., 2022). The DEGs associated with DAMs included four genes among the MYB family of transcription factors (*Csa1G071840*, *Csa2G403160*, *Csa4G638510*, and *Csa1G586860*) and one for a bZip transcription factor (*Csa2G403160*). The MYB transcription factor coding gene (*Csa4G638510*) activated transcription of the auxin response gene IAA19 in response to auxin. Shikimic acid produces phenylalanine under the action of the R2R3MYB transcription factor coding gene (*Csa1G071840*), which can prompt the shimoic acid pathway to regulate volatile benzene and phenylpropane-activated EPSPS, ADTI, CFTA, and CCoAOMT1 genes (Van Moerkercke et al., 2011; Shaipulah et al., 2016). Salicylic Acid (SA) is an important phenolic acid compound. In recent years, the PBS3 gene was identified as the most critical enzyme encoding gene in the SA biosynthetic pathway (Rekhter et al., 2019). SA is an important plant disease resistance mediator. In our pathway, SA is synthesized by the bZip transcription factor encoding gene (*Csa2G403160*). This gene can mediate auxin and salicylic acid induced transcription in cauliflower Mosaic virus, and inter-act with NPR1 to induce systemic acquired resistance in plants (Després et al., 2003). At the same time, SA can be reached by Salicylic Acid 5-Hydroxylase and participate in pathogen sensitivity. Moreover, SA is widely expressed from seedling to adult stage (Zhang et al., 2017; Rekhter et al., 2019; Torrens Spence et al., 2019). SA is hydroxylated by the MYB transcription factor coding gene (*Csa1G586860*) to form diisooctyl phthalate and bis (2-ethylhexyl) phthalate. This gene activates SA-mediated defense and resistance to pathogens (Shim et al., 2013). In summary, these transcription factor coding genes may be related to FW resistance genes.

### 4.3 Antimicrobial effect of phthalate derivatives

Phthalates are widely known as polymer materials in plasticizer. However, Phthalate compounds have been found in the secondary metabolites of plants, animals and micro-organisms since 1967 (Zhang et al., 2018; Roy, 2020). *Eichhornia crassipes* can produce mono-(2-ethyl hexyl) phthalate, which presents bioactivity against *Chl. Vulgaris* (Sultan et al., 2009). Traditional medicinal plants produce an abundance of phthalate compounds with a variety of activities. These compounds isolated from the hairy vetch buds of *Viciavillosa Roth* showed inhibitory effects against phytopathogenic strains such as *Rhizobium Cheonan* 493 and *Bacillus subtilis* (Islam et al., 2013). Phthalate compounds isolated from *Sysimbrium officinale* showed broad-spectrum antimicrobial activities against Gram-positive and pathogenic fungi at a concentration of 0.5 mg/mL (Blažević et al., 2010). Phthalates were also detected in the soil of tomatoes grown after biosolids application and radishes grown after composting (Mo et al., 2008; Sablayrolles et al., 2013). In addition, 13 different phthalates were isolated from the fruits of Acanthopanax sessiliflorus (*Araliaceae*) (Asilbekova et al., 1985). In 2020, N. Kumari et al. reported on the isolation of dibutyl phthalate (5) as secondary metabolites of an actinomycetes strain grown on an actinomycete isolation agar. However, in the same study, tert-butylcalix arene, a synthetic product, was also found to be a secondary metabolite of the actinomycete strain (Kumari et al., 2020). Phthalic acid has been found in a number of plant extracts, such as in an ethyl acetate extract of *Bridelia ovata* and ethanolic extracts of licorice (*Glycyrrhiza glabra*) leaves, sometimes in concert with phthalates (Mohan and Anand, 2019; Poofery et al., 2020).

In this study, metabolic analysis identified two phenolic acid compounds, diisooctyl phthalate and bis(2-ethylhexyl) phthalate that were accumulated in a significantly higher quantity in NR than in NS at 4 dpi (**Supplementary Tables S1, S2**). *In vivo* assays suggested that these compounds had inhibitory effects on *Foc* growth (**Figure 4**). The root exudates of Barnyard grass (*Echinochloa crusgalli*) contain diisooctyl phthalate, which reduces the germination and growth of monocotyledonous plants, lettuce, and rice (Xuan et al., 2006). Diisooctyl phthalate is secreted from the water hyacinth (*Eichhornia crassipes*), and possesses strong inhibitory effects on *Chlorella vulgaris* (Liang et al., 2008). Bis (2-ethyl hexyl) phthalate can be produced by the strain of the fungus *Cladosporium* sp. F14 (Qi et al., 2009). Bis(2-ethylhexyl) phthalate isolated from the flower of *Procera gigantea* was found to be active against the Gram-positive *bacteria staphylococcus aureus, bacillus subtilis, btreptococcus equosemens* and *sarcina lutea* and against the Gram-negative *bacteria closteridium perfringens, pseudomonas aeruginosa* and *shigella dysenteriae* (Habib and Karim, 2009; Sayed, 2013).

## 5 Conclusions

In this study, based on metabolic and transcriptional data from the phenotype investigation of NILs, the genes and metabolites may relate to cucumber fusarium wilt resistance were determined. The results showed that phenolic acids and flavonoids were the primary DAMs between NR4d and NS4d, and phenolic acids and flavonoids metabolites could greatly improve resistance to FW. These metabolites will be tested for field control of cucumber FW, which will provide a new direction for field control. These results lay an important foundation for further complementing and improving the research on cucumber resistance mechanisms, provide references for the screening of related genes for fine-mapping, and promote the breeding of cucumber FW resistance lines. However, other key genes related to FW resistance require further exploration and verification.

## Data availability statement

The datasets presented in this study can be found in online repositories. The names of the repository/repositories and accession number(s) can be found below: https://www.ncbi.nlm.nih.gov/, PRJNA916850.

## Author contributions

KY: Conceptualization, Data curation, Formal analysis, Investigation, Methodology, Software, Validation, Visualization, Writing – original draft, Writing – review & editing. GZ: Data curation, Formal analysis, Investigation, Methodology, Software, Validation, Visualization, Writing – review & editing. CC: Conceptualization, Data curation, Funding acquisition, Project administration, Resources, Writing – review & editing. XL: Conceptualization, Resources, Validation, Writing – review & editing. LW: Conceptualization, Software, Validation, Visualization, Writing – review & editing. FZ: Conceptualization, Data curation, Formal analysis, Software, Visualization, Writing – review & editing. ZL: Conceptualization, Supervision, Validation, Visualization, Writing – review & editing. HC: Conceptualization, Funding acquisition, Project administration, Resources, Supervision, Validation, Visualization, Writing – review & editing.
