# Supplementary material for: Joint metabolomic and transcriptomic analysis identify unique phenolic acid and flavonoid compounds associated with resistance to fusarium wilt in cucumber (Cucumis sativus L.)
Source: Front Plant Sci. 2024 Aug 7;15:1447860. doi: 10.3389/fpls.2024.1447860 (PMC11335689; doi:10.3389/fpls.2024.1447860)
Supplement: Supplementary file 1 [file DataSheet_1.docx]

Supplementary Material

# Supplementary Figures and Tables

## Supplementary Figures


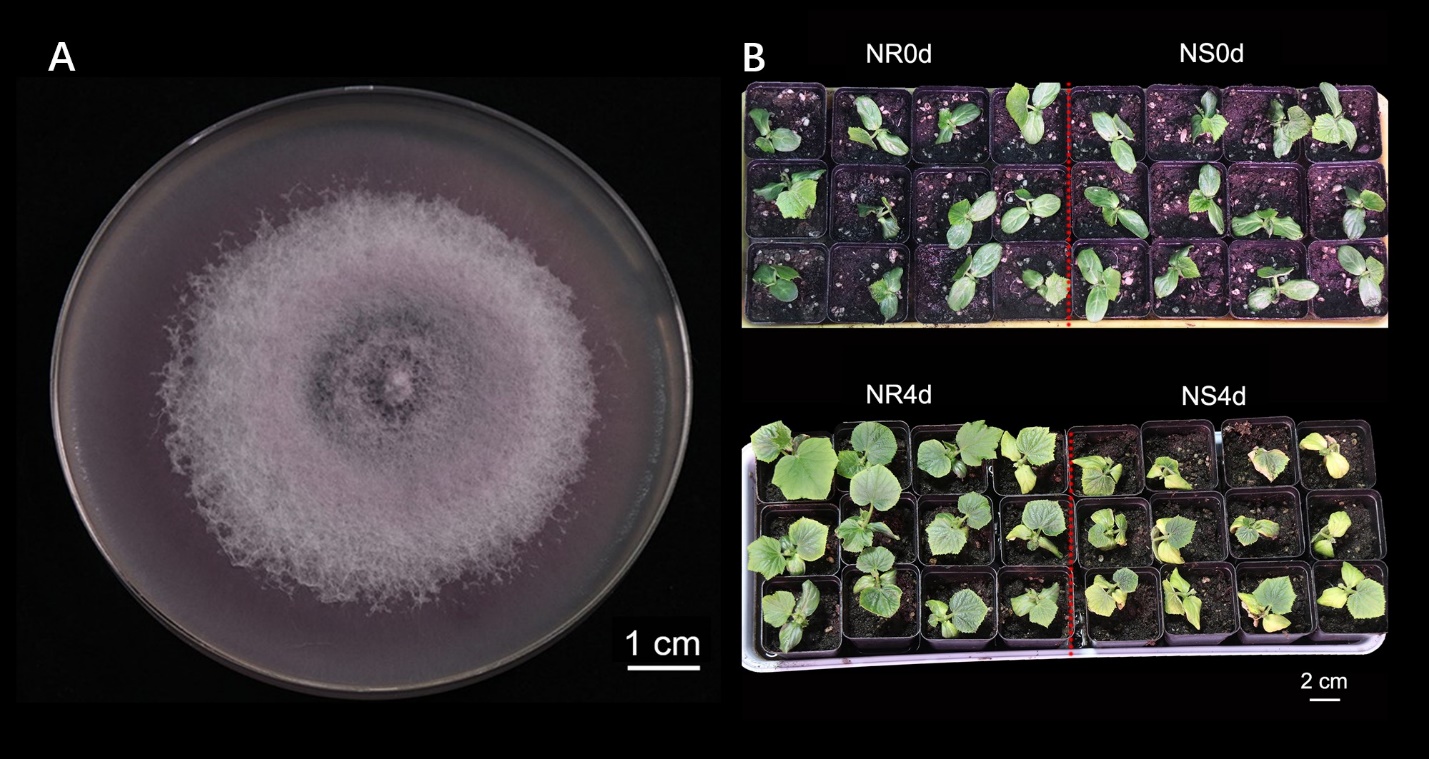


**Supplementary Figure 1.** *Foc* inoculation responses in seedlings of NR and NS NILs. (A) Culture of the *Foc* pathogen isolated from the soil in the plastic greenhouse used for FW resistance screening. (B) At seedling stage, 4 days after *Foc* inoculation, chlorosis of the cotyledons and first true leaves are clearly visible in NS but not in NR.


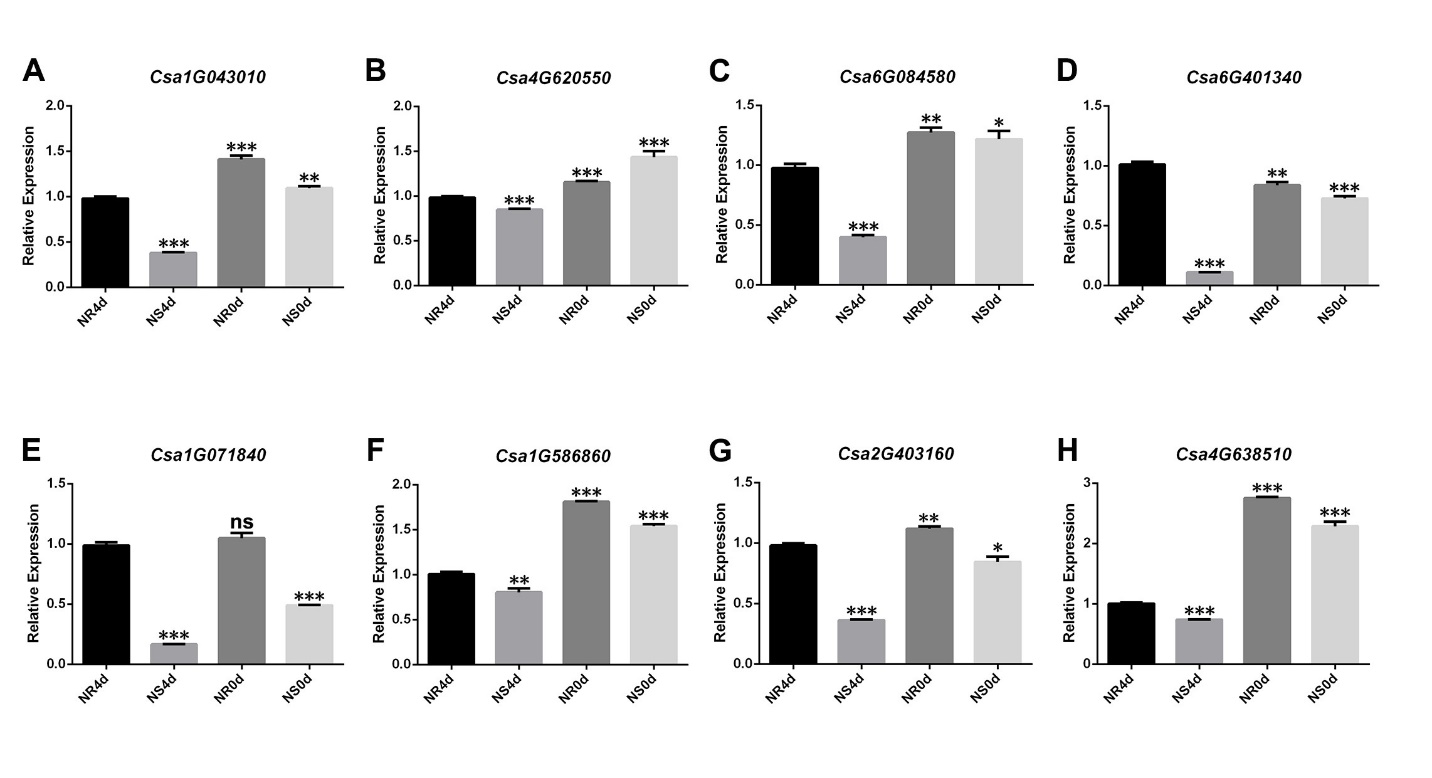


**Supplementary Figure 2.** Validation of selected DEGs with qRT-PCR. Bars are means of three replicates ± SD. Asterisks (*) indicate the statistical significance of the difference between the experimental groups (NS4d, NR0d and NS0d) and the control group (NR4d). *P < 0.05, ** P < 0.01, *** P < 0.001, ns = not significant.


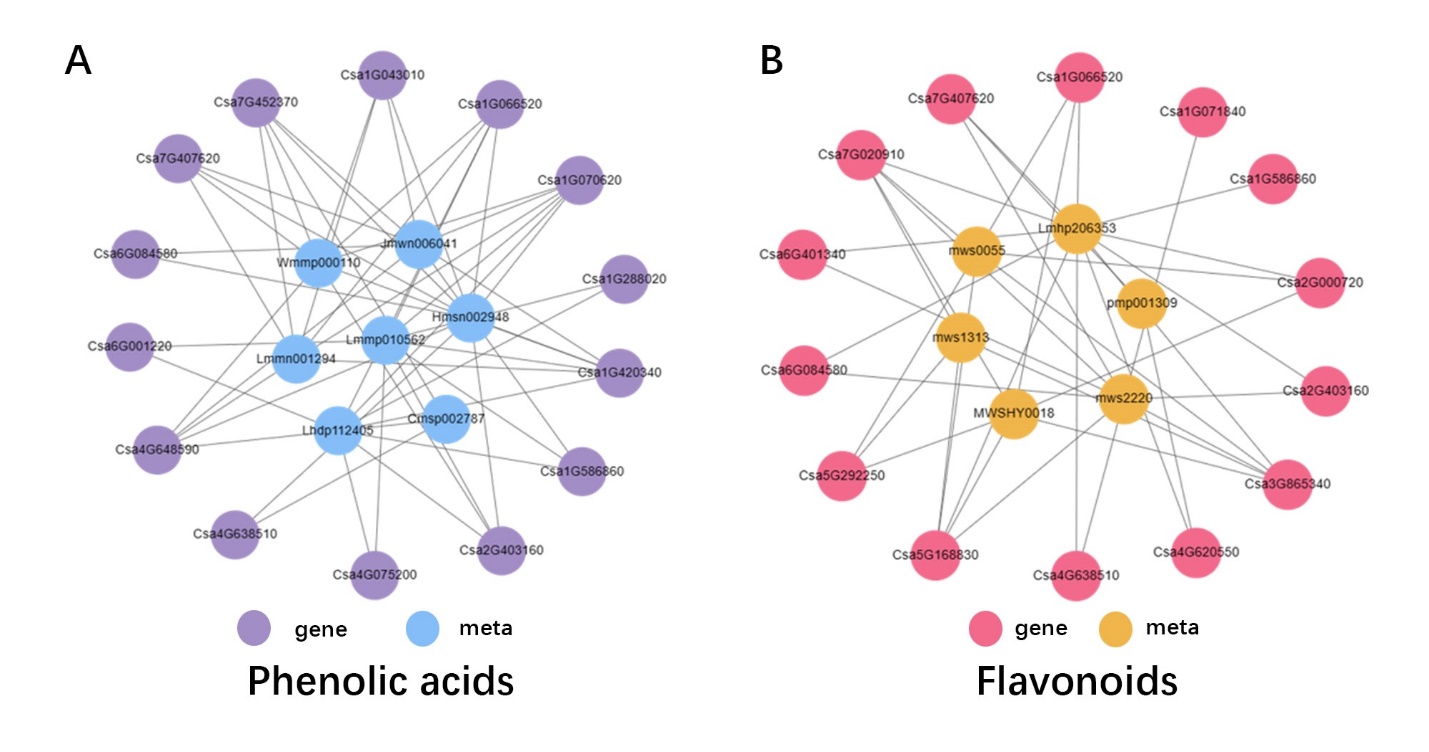


**Supplementary Figure 3.** Network analysis of DEGs and DAMs. (A) Correlation network of DEGs and DEMs involved in Phenolic acids. purple circles indicate genes, and blue circles indicate metabolites. Determined by a Pearson correlation coefficient > 0.8 or < −0.8, respectively. (B) Correlation network of DEGs and DEMs involved in flavonoids. Red circles indicate genes, and yellow circles indicate metabolites determined by a Pearson correlation coefficient > 0.8 or < −0.8, respectively.
